# Assessing quality of online learning platforms for in-service teachers’ professional development: The development and application of an instrument

**DOI:** 10.3389/fpsyg.2022.998196

**Published:** 2022-10-07

**Authors:** Jing Zhang, Bing Wang, Harrison Hao Yang, Zengzhao Chen, Wei Gao, Zhi Liu

**Affiliations:** ^1^School of Education, Jianghan University, Wuhan, China; ^2^National Engineering Laboratory for Educational Big Data, Central China Normal University, Wuhan, China; ^3^School of Education, State University of New York at Oswego, Oswego, NY, United States; ^4^School of Education, Central China Normal University, Wuhan, China

**Keywords:** teacher education, online learning, quality evaluation, user experience, training platform

## Abstract

To help optimize online learning platforms for in-service teachers’ professional development, this study aims to develop an instrument to assess the quality of this type of platforms on teacher satisfaction. After reliability and validity tests and expert empowerment, the 27-item instrument was formed. Based on the information systems (IS) success model, this instrument was designed to measure teacher perceptions of the quality of online learning platforms from three dimensions, namely, content quality, technical quality, and service quality. Moreover, the developed instrument was used to analyze the effects of the National Teacher Training Platform amid the COVID-19 outbreak in China. The findings revealed that the improvement of the platform’s style, tool function, operating efficiency, and teaching methods could enhance teachers’ experience of online training.

## Introduction

Over the years, education reform and teacher training initiatives have worked hard to build and promote scalable, sustainable online communities for education professionals (Schlager and Fusco, 2003). One of these types of communities, online learning platforms for teacher professional development (TPD), has garnered widespread attention and has been growing rapidly for its capacity and flexibility to support teachers to continuously reflect, learn, and act to augment their practice throughout their teaching careers. Online learning platforms make teacher training and self-development feasible and more convenient in terms of time and space. A quality online teacher learning platform is an essential guarantee for positive teacher learning effects (Pengxi and Guili, 2018). Online technology can help supply high-quality TPD when it is suitably integrated into the learning platform. However, using online technology as a “quick fix” or integrating it into a platform without a clear purpose will not result in the desired changes in teaching and learning outcomes (Cobo Romani et al., 2022). Schlager and Fusco (2003) contended that focusing solely on online technology as a means of delivering training and/or creating online networks puts the cart before the horse by ignoring the Internet’s even greater potential to support and strengthen local communities of practice in which teachers work. The design and delivery of high-quality TPD warrant an understanding of the applicable technology, resources required, and teachers’ needs. Previous research suggested that to exert the greatest impact, professional development must be designed, implemented, and evaluated to satisfy the needs of particular teachers in particular settings (Guskey, 1994). Thus, the teachers’ perceptions of online learning platforms for TPD have become an issue of great importance, especially with regard to the quality of these platforms. Obtaining and analyzing teachers’ perceptions can help trainers, administrators, platform designers, and technologists better use and improve online learning platforms for TPD, thereby helping teachers acquire knowledge and skills more effectively. As Greenberg (2009, p. 2) pointed, “without a programmatic understanding of best practices and methods of anticipating potential roadblocks, far too many initiatives may falter or fail.”

So far, despite the availability of some instruments to measure users’ perception of online platforms or websites in general, there are few instruments specifically for teachers’ perception of the quality of online training platforms. Without appropriate measurement, some online learning platforms might not be used effectively to support TPD. To address this significant issue and research gap, this study aims to: (i) develop a teacher perception scale of the quality of online learning platforms for TPD; and (ii) apply the developed scale to evaluate the National Online Teacher Training Platform in Central China.

## Study design

**FIGURE 1:**
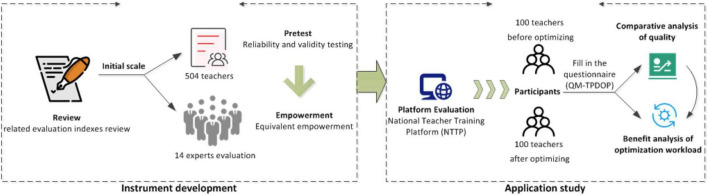
Study design.

Overall, this study contained two parts, instrument development and application study. As shown in Figure 1, the first part included four steps of the instrument development: conceptual framework and related works, initial scale, preliminary test, and expert evaluation empowerment. Then, the developed instrument was used to validate and evaluate the optimization effect of the online learning platform for teachers. The second part of this study included the following steps: pre-analysis of data, platform quality comparison, and benefit analysis of platform optimization.

## Instrument development

### Conceptual framework and related works

**FIGURE 2:**
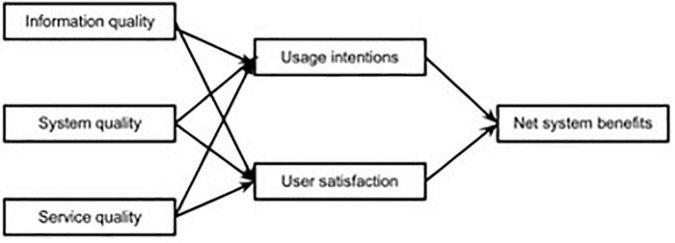
Information systems (IS) success model.

One of the leading models for measuring information systems (IS) is the IS success model, which aims to provide a thorough understanding of IS success by elucidating the relationships among the critical success factors frequently considered when assessing IS. The IS success model was initially developed and later revised by DeLone and McLean, 1992, 2002, 2003 in response to input from other researchers. As shown in Figure 2, in the IS success model, the three dimensions of quality (information or content quality, system quality, and service quality) directly affect usage intentions and user satisfaction, and, consequently, net system benefits. *Information quality*, also known as content quality, denotes the quality of the information or content that a system can store, deliver, or produce in terms of completeness, relevance, and consistency. *System quality*, also known as technical quality, denotes the quality of the system in terms of functionality, usability, efficiency, and portability. *Service quality* usually denotes the quality of support provided to the users, including reliability, responsiveness, assurance, and empathy. So far, the IS success model has been adopted widely by existing studies to measure different types of information systems, including online learning systems and websites (Lin, 2007). Table 1 presents some main relevant instruments based on one or all of the quality dimensions. Based on the IS success model and previous related research, this study develops an instrument specifically on online platforms for teachers’ professional development. Specifically, this study attempts to identify key indicators from technical quality, content quality, and service quality to measure the effectiveness of online learning platforms for teachers’ professional development on teachers’ experience.

**TABLE 1:** Main instruments of online learning platforms and websites.

Author	Object	Sample/target	Measurement	Indicators	Category		
					Content quality	Technical quality	Service quality
Hassanzadeh et al., 2012	Measurement of e-learning systems success model (MELSS)	33 experts, and 369 instructors, students, and alumni from five universities	5-point scale, quantitative	Technical system quality, educational system quality, content and information quality, service quality	√	√	√
ÖZkan et al., 2020	Evaluation criteria of search engine optimization (SEO)	70 Turkish industrial engineering departments’ websites	Quantitative form, qualitative/quantitative	Performance, design, content, meta tags, backlink, technical		√	
Velasquez and Evans, 2018	A website’s assessment spreadsheet protocol	46 postgraduate students	A spreadsheet protocol, quantitative	Accessibility of websites, available online resources, library staff’s responses	√	√	√
Liu et al., 2011	Evaluation criteria for Web usability of English learning websites	160 university students and seven learning technology and English teaching experts	The derived criteria combined with a checklist, qualitative/quantitative	Web usability, learning materials, functionality of assisting language learning, technology integration, and learner preferences	√	√	√
Yang and Chan, 2008	Set of evaluation criteria for English learning websites	Eight students and eight English teachers selected from junior high schools, 17 experts	4-point scale, qualitative/quantitative	General information, integrated English learning, listening, speaking, reading, and writing	√		

#### Content quality

Content is the core of online learning platforms, including information and resources for learning and practice. Yang and Chan (2008) focused on the content quality in the evaluation of English learning websites and prepared a differentiated evaluation of the general content, professional learning content, and exclusive training content of each website, highlighting the authority and practicality of content. In addition, Devi and Verma (2018) investigated libraries in general, as well as service information and different types of professional books, stating that attention should also be paid to the design of learning methods and strategies besides the quality of the content itself. Desjardins and Bullock (2019) claimed that only when teachers experience the problem-based learning mode in online training, their learning stays at the theoretical level with no conflicts and contradictions. Fuentes and Martínez (2018) analyzed different evaluation frameworks for English learning platforms and proposed an assessment list, including multimedia, interactive and educational content, and communication items, covering the assessment of teaching content, teaching methods, and strategies.

With the progress of teachers’ online activity, many online learning platforms are promoting both teacher tuition and practice, providing teachers with curriculum research, materials, and resources for their teaching practice. Thus, the content quality of this study denotes the quality of online learning courses and teaching-related professional resources. Regarding specific sub-dimension design, we integrated previous research on evaluating the teaching content and method strategies with the teaching effect suggested by experts. Then, content quality indicators were constructed from three aspects: resource, method, and effectiveness. Of note, resource evaluation highlights the authority and practicality of the content. Method evaluation focuses on diversity and individual motivation. Teaching effect evaluation emphasizes the impact of learning on Teachers’ teaching practices.

#### Technical quality

Regarding evaluating the platform’s technical quality, most studies evaluated the two general aspects of the functional effectiveness and technical aesthetics of online platforms or websites and then proposed enhancement in operation technology and interface design. Reportedly, improving online platforms helps to increase user satisfaction and user application continuity (Ng, 2014; Laperuta et al., 2017; Liu et al., 2017; Manzoor et al., 2018; Mousavilou and Oskouei, 2018). Santos et al. (2016, 2019) followed the five principles, namely, multimedia quality, content, navigation, access speed, and interaction in graphic design, to measure user satisfaction in online learning websites. They revealed that usability and navigation were the two evaluation subindicators most preferred by users.

Besides the basic usability aspect, the technical quality of the online platform is the operation’s efficiency and the users’ stickiness in the application process. For example, ÖZkan et al. (2020) aimed at the rapid upsurge of resource retrieval, assessing the retrieval quality of academic online learning platforms from the standpoint of performance, design content, meta-tags, backlinks, and other indicators. Pant (2015) examined the quality of the central science library’s websites primarily from the aspects of efficiency, satisfaction, and accessibility. The evaluation items included the smooth and fast operation of web pages, the ease of use of functions, and the reliance and trust of website services after application. Hassanzadeh et al. (2012) investigated the success factors of digital learning systems and proposed that user loyalty is a factor affecting the system’s success. Loyalty signifies users’ dependence on the platform and the willingness to recommend the platform. This study considered that teachers are different in sensitivity to technical efficiency, interface design, and overall design. Thus, in terms of assessing the rationality and effectiveness of online technology, we combined users’ perception of the development speed of the platform and users’ willingness to recommend the platform. Overall, the evaluation of online teacher learning platforms is conducted from the aspects of effectiveness, style, and development in terms of technical quality.

#### Service quality

Service quality is one of the key factors influencing learner satisfaction. Examining the quality of digital learning services, user satisfaction, and loyalty, reported that organizational management and learning support, as well as course quality, could affect learners’ satisfaction. Online learning services included similar human services, resources, and tool services. Velasquez and Evans (2018) focused on the public library website staff’s response time to users in terms of service quality and revealed that user satisfaction with the website could be increased by refining the staff’s service. Devi and Verma (2018) included webpage emergency response and tools as one of the quality evaluation items when assessing library websites. Fuentes and Martínez (2018) involved learning support tools and learning resources in assessing website quality when evaluating English learning websites. Moreover, the evaluation of learning services should reflect life-oriented characteristics. Using a design-based research method to propose an evaluation of English learning websites, Liu et al. (2011) analyzed those websites from the aspects of network availability and learning materials, as well as functions that assist language learning, technology integration, and learner preferences. The evaluation indicator system not only accentuates the platform that provides the systematic learning of course content for teachers but also focuses on providing extensive resources and functional applications. Regarding the evaluation of resources and functions, the indicator focuses on the platform’s richness and diversity, while it focuses on personalization in terms of learning services.

With the diversification of online learning methods for teachers and personalized online learning trends in data analysis, the formulation of service quality indicators comprehensively considers manual services, such as answering questions and providing guidance. In addition, developing data analysis and diagnosis services is included, as well as supporting teachers’ teaching, function modules, and apps for behaviors like research, discussion, and reflection. Regarding the evaluation of service items, this study focused on the effectiveness of individualized assistance, as well as the relevance and availability of related tool functions and services.

### Initial scale

We constructed the teacher perception scale of the quality of online learning platforms for TPD (TPS-Online-TPD), including 27 question items (Table 2). Moreover, the scheme provided the meaning described by each indicator.

**TABLE 2:** The dimensions and indicators of TPS-Online-TPD.

Dimensions	Indicators	Description
Technical quality	Efficacy	Speed and efficiency of platform operation, page jump, data, and resource transfer.
	Style	Reasonable and aesthetic level of platform function, interface, font, color.
	Development	The growth rate of users and resources of the platform; trust and recommendation intention of users with regard to the platform.
Content quality	Resources	The authority, reliability, and rationality of the courses and teaching resources.
	Effectiveness	The influence of network learning on the teacher’s teaching theory and practice.
	Method	The reasonableness, diversity, and stimulating nature of the teaching methods and strategies.
Service quality	Help	Access to help and response time when users encounter problems.
	Functional tools	The utility and richness of the tools and functions that support teacher learning and long-term professional development.
	Guidance	The degree of user satisfaction and personalization of the learning guidance, answering of questions, and other support services provided by the platform.

Technical quality measured the platform performance and the rationality of the functional interface design. The technical quality evaluation included the evaluation of users’ dependence on the platform and the development of perception, including the following: efficacy (3 items), style (4 items), and development (4 items). An example is “I think the overall layout of the platform is reasonable.”

Content quality measured the quality of all content, including online courses and teaching resources. The evaluation involved not only the authority, rationality, and reliability of the resource itself but also the learning process and results, including resources (4 items), effectiveness (2 items), and method (3 items). An example is that “The teaching content and resources provided on the platform are authoritative.”

Service quality measured the rationality and effectiveness of platform support services. It focused on the service quality that a platform is able to deliver, including: help (2 items), functional tools (3 items), and guidance (2 items). An example: “The analysis of learning data provided by the platform is very good for my learning.”

### Preliminary test

A preliminary test TPS-Online-TPD containing 27 items was developed and distributed for online participation. The target population was primary and secondary school in-service teachers who had just completed a period of online training. They were required to complete evaluations of the learning platform they had used, based on their real experience. An exploratory factor analysis was performed on the recovered questionnaire with SPSS22, and the reliability and validity of the results were verified.

#### Participants

Participants were in-service teachers who completed their professional development training on different online learning platforms hosted by the National Teacher Training Center at Central China Normal University in 2018. A total of 567 questionnaires that contained items of TPS-Online-TPD were collected online. After excluding the high rate of identical answers and incomplete or blank answers, 504 valid questionnaires were obtained, with the efficiency of the questionnaire at 88.89%. Of 504 participants, 77.4% were teachers in primary schools, and 22.6% were teachers in middle schools. In addition, 41.2% of teachers held senior titles in total. Regarding gender, 71.2% are women, while 28.8% are men. For the length of their teaching careers, 3.9% of respondents had been teaching for >3 years, 16.3% from 3 to 10 years, and 73.8% for >10 years. The age information range of participants was as follows: 14.3% aged <30 years, 43.3% aged 30–39 years, 34.9% aged 40–49 years, and 7.5% aged >50 years. Finally, 11.3% of the responding teachers had no experience in online training.

#### Validity and reliability

First, the questionnaires were classified to some extent using exploratory factor analysis. We used the principal factor analysis, and Varimax performed factor rotation in the factor analysis process. The cumulative variance explanation rate was the proportion of the variance due to all factors to the total variance, suggesting the total influence of all factors on the dependent variable. Typically, the cumulative variance explanation rate should at least be >50%; while >70% is better, >85% is excellent. According to the cumulative variance interpretation rate, three factors with eigenvalues >1 were extracted. Table 3 shows that three common factors have eigenvalues >1; the total variance interpretation rate for the three factors is 71.523%. Hence, most of the factors were considered to have been covered. Thus, the original three-dimensionality is retained as a common factor.

**TABLE 3:** Eigenvalue and accumulative rate of factor analysis.

Principal component	Initial eigenvalues			Rotation sums of squared loadings		
	Eigenvalue	Explained variance (%)	Accumulative rate of contribution (%)	Eigenvalue	Explained variance (%)	Accumulative rate of contribution (%)
1	26.42	64.43	64.43	10.59	25.84	25.84
2	1.76	4.28	68.71	9.88	24.09	49.92
3	1.15	2.81	71.52	8.86	21.60	71.52
4	0.77	1.87	73.40			
5	0.68	1.66	75.06			

The load factor was the load of a variable on a common factor, which, in turn, reflected the relative importance of the variable on the common factor. Typically, the load factor after rotation must be >0.71 to be excellent, >0.63 to be very good, and >0.55 to be good. As shown in Table 3, the load factor of only one question in this questionnaire was <0.63, while that of all questions was >0.55, indicating that the questionnaire had a high degree of correspondence with the dimensions, and all 27 questions could be reserved. Supplementary Appendix 1 lists all questions.

Then, we tested the reliability of the questionnaire using the Cronbach coefficient for the 27 selected items. Usually, a Cronbach coefficient of 0.70 is credible, while 0.70–0.98 indicates high reliability. Table 4 demonstrates that the reliability coefficients of the three dimensions are all >0.70, with some even >0.95; the overall reliability coefficient of the questionnaire reached 0.98, indicating that the questionnaire had excellent internal consistency. Next, the KMO test statistic of the screened questionnaire was analyzed. Of note, the KMO test statistic is an index used to compare the simple correlation coefficient and the partial correlation coefficient between variables. The closer the KMO value is to 1, the stronger is the correlation between variables, and the more suitable the original variables are for factor analysis. Of note, a KMO value of 0.90 indicates excellent (marvelous), while 0.8 indicates good (meritorious), and 0.70 indicates middling. The KMO value of this questionnaire was 0.98, indicating that the questionnaire was highly suitable for factor analysis. Finally, we performed a factor analysis on the screened questionnaire. The factor loading coefficients of each question are in the table, and the corresponding factors were 0.61–0.80, indicating that the questionnaire had excellent structural validity.

**TABLE 4:** Analysis of the final questionnaire’s validity and reliability.

Measure	Item	Factor loading	α	KMO
Technical quality	T1. The resources on the platform are growing fast.	0.74	0.96	
	T2. I would like to recommend this platform to friends and colleagues.	0.76		
	T3. I am delighted with the way the tools/sections on the webpage open, run, and jump.	0.80		
	T4. The webpage on the platform runs smoothly.	0.68		
	T5. The running speed of webpages on the platform and the uploading and downloading of resources are fast.	0.68		
	T6. It is very efficient to communicate and share resources through the platform.	0.71		
	T7. I think the overall page layout of the platform is reasonable.	0.71		
	T8. The colors and fonts of the platform page are well designed.	0.69		
	T9. The functional navigation of the webpages on the platform is clear.	0.72		
	T10. The colors and fonts of the platform pages are well designed.	0.67		
	T11. The functions and resources of the platform can support my long-term use of the platform.	0.67		
Content quality	C1. On the platform, I can retrieve many resources that I need for teaching.	0.71	0.95	
	C2. I trust the learning content and resources provided by the platform.	0.74		
	C3. The teaching content and Q and A provided on the platform are authoritative.	0.71		
	C4. The content on the platform is in line with the needs of our teachers’ learning and development.	0.75		
	C5. The content of this learning platform is closely related to my teaching practice.	0.78		
	C6. After stages of learning, my teaching concepts and ideas have changed.	0.78		
	C7. The teaching content on the platform and the teacher’s teaching are exciting and help me maintain my continuous enthusiasm for learning.	0.68		
	C8. The learning method of the course is suitable for my professional development needs.	0.68		
	C9. I am satisfied with the variety of learning methods available on the platform.	0.61		
Service quality	S1. There are clear channels on the platform to help with problems.	0.67	0.96	
	S2. The platform has fast support services.	0.69		
	S3. The functional tools provided on the platform are very useful.	0.72		
	S4. The functional tools available on the platform are plentiful.	0.75		
	S5. The analysis of learning data provided by the platform is very beneficial to my learning.	0.67		
	S6. The platform provides personalized services, such as learning strategies and learning guidance.	0.71		
	S7. The quality of support services provided by the platform, such as answering questions and guidance, is high.	0.68		
Total			0.98	0.98

### Expert evaluation empowerment

As indicators are of different importance, we used the expert assessment method to empower the indicators at all levels. The data were collected through an online questionnaire and calculated with an equal weight evaluation method. First, we obtained the weights assigned to the indicators by experts through a questionnaire survey. Thus, the average overall score of each indicator item was calculated per the ranking of all the fill-in options, reflecting the overall ranking of the indicator. The calculation method is:


(1) $$S_{\text{i}}=\frac{\sum \left(F_{i\text{j}}\;\times \;W_{\text{ij}}\right)}{F}$$

where *S*_*i*_ is an average overall score of option *i*; *F*_*ij*_ denotes the times of option *i* in position *j*; *W*_*ij*_ denotes the weight of option *i* in position *j*; *F* denotes the times fill in this question.

The option average overall score was further processed to obtain the platform indicator weight value. The calculation formula is as follows:


(2) $$k_{\text{i}}=\frac{S_{i}}{S_{n}}\;\times \;100\%$$

where *k*_*i*_ denotes the weight of option *i*; *S*_*i*_ denotes the average overall score of option *i*; S_*n*_ denotes the sum of the average overall scores of all options in the dimension.

The ranking questionnaires were distributed to 14 experts, including teachers and subject supervisors from primary and secondary schools and eight instructors and advisors from universities, all of whom specialized in teacher mentorship, teacher preparation, and online learning programs. They have about an average of 23 years working experience. Supplementary Appendix 2 provides the experts’ primary background information. According to the formula above, the weights of the indicators at all levels of the platform were calculated, as shown in Table 5.

**TABLE 5:** Indicator weights.

Dimensions	Weight	Indicators	Weight
Technical quality	0.29	Efficacy	0.42
		Style	0.25
		Development	0.33
Content quality	0.49	Resources	0.42
		Effectiveness	0.30
		Method	0.28
Service quality	0.23	Help	0.36
		Functional tools	0.29
		Guidance	0.36

## Application study

### Data analysis

#### Study process

To validate the practicability of TPS-Online-TPD, we applied it to the annual evaluation of the National Online Teacher Training Platform in Central China in November 2019 and July 2020, respectively, which was before and after the lockdown of Wuhan. The Pearson correlation test and independent-sample *t*-test were conducted to ensure the data from two different groups of teachers were available for comparison before conducting a comparative analysis. Of note, the difference between the two evaluations could represent platform improvement. To further investigate the efficiency of each part of the improvement work, we further calculated the ratio between quality improvement and workload, to comprehend which improvement work could attain the fastest improvement of user satisfaction with the least workload.

#### Participants

We commissioned the development agency of the training platform; they invited 200 teachers (100 in November 2019 and 100 in July 2020) to fill in the questionnaires carefully in the form of a formal invitation letter through the administrative personnel of the school. All invited in-teachers were from primary and secondary schools in urban areas who had online learning experience before. We checked the collected questionnaires and found that all questionnaires were available in terms of time spent answering questions and repetition rates of the same answers.

#### Data preprocessing

We performed the following analyses to ensure that the data collected from the two evaluation surveys were effective for comparative analysis. First, the Pearson correlation test was conducted on the correlation between scores measured in 2019 and 2020 and the background factors of samples. The significance coefficient *P*-value obtained was >0.05, as shown in Table 6, suggesting that the correlation was not significant; thus, the personnel background factors would not affect the scoring results, and the data were usable. Then, we conducted an independent-sample *t*-test. In addition, Levene’s test for equality of variances was not significant, and the corresponding *P*-value was <0.05, as shown in Table 7, indicating a significant difference between the two measurement results and that the results could be compared.

**TABLE 6:** Correlation between the total platform score and variables.

Version of platform	Coefficient	Variables				
		Seniority	Age	Title	Online training experience	Gender
2020	Correlation	−0.01	−0.47	0.03	−0.05	−0.07
	Significance	0.91	0.64	0.80	0.66	0.52
2019	Correlation	−0.09	−0.06	−0.07	−0.12	0.13
	Significance	0.36	0.58	0.48	0.23	0.20

**TABLE 7:** Independent-sample *t*-test of the two versions of the platform.

Version of platform	Mean	SD	Difference of mean	SE	*P*	95% Confidence intervals		Levene’s test for equality of variances	
						Upper limit	Lower limit	*F*	Significance
2020	4.20	0.42	0.14*	0.07	0.04	0.005	0.27	3.12	0.079
2019	4.06	0.53							

*P < 0.05.

## Results

Table 8 presents the results of the two weighted measurements. The scores of all indicator items were higher in 2020 than those in 2019, showing that optimizing online teacher learning platforms amid COVID-19 improved user experience in various aspects. According to the data collected during the COVID-19 pandemic, online learning was teachers’ only professional development method. Thus, the influence of other forms of professional learning for teachers can be excluded from the study based on the data collected during this period.

**TABLE 8:** Comparison of workload, improvement, and improved efficiency.

Dimensions/key indicators	2019	2020	Workload	Improvement	Improved efficiency
Technical quality	4.15	3.97	65	0.18	28.15
Content quality	4.25	4.17	58	0.09	14.66
Service quality	4.18	4.02	88	0.16	18.18
Efficacy	4.13	3.87	37	0.26	70.27
Style	4.19	4.05	10	0.15	146.00
Development	4.16	4.04	18	0.12	65.00
Resources	4.25	4.15	41	0.11	25.61
Effectiveness	4.28	4.21	–	0.07	–
Method	4.22	4.15	14	0.07	50.00
Help	4.18	4.01	25	0.17	66.00
Functional tools	4.22	4.07	18	0.15	81.67
Guidance	4.17	4.01	45	0.17	36.67
Total	4.21	4.08	211	0.13	6.16

The more significant the difference measured, the higher the improvement. We observed that the improvement in technical and service quality were the most significant, followed by the improvement in content quality. Overall, the improvement in webpage operation efficiency was the most significant in technical quality. The improvement in the resource quality in terms of content quality was relatively high, and so was the improvement in assistance and guidance in service quality, which were more significant than the improvement in functional tools.

After comparing the weighted average scores of the two measurements, we further combined the National Online Teacher Training Platform Operation Company’s annual improvement efforts in each module to elucidate the benefits and effects of platform improvement. The calculation formula is shown in Eq. 3, and Table 7 presents the calculation results. The bigger the input–output ratio, the higher the benefit of the improvement.


(3) $$P_{\text{n}}\;=\;\frac{A_{n}\;-\;A_{n\,m}}{L_{n}}\;\times \;C$$

where *P*_*n*_ denotes efficiency improvement in dimension *n*; *A*_*n*_ denotes the weighted average score of dimension *n* this year; *A*_*nm*_ denotes the weighted average score of dimension *n* last year; *L*_*n*_ is the workload of dimension *n*; *C* denotes the fixed rounding factor with a value of 10,000.

As shown in Table 8, it is apparent that the degrees of improvement and improved efficiency in technical quality were higher than those of the quality of service and content. Among the key indicators, the improvement in the interface style was the most efficient. Regarding the optimization in content quality, improvement in the teaching method could lead to high efficiency. The improvement of each dimension of service quality was relatively balanced, and the improvement of the platform function tools was the most efficient.

## Discussion and conclusion

Based on the IS success model and previous relevant research, this study developed TPS-Online-TPD, which provides a set of key indicators for assessing the quality of online learning platforms for teacher professional development from three aspects: technical quality, content quality, and service quality. TPS-Online TPD is further used to analyze the optimization of the National Teacher Training Platform amid the COVID-19 outbreak in China. By comparing the quality evaluation of the platform before and after the lockdown of Wuhan. The findings revealed two main points. First, the optimization of the platform’s technical quality and service quality can better improve teachers’ learning experience, compared with the optimization of the platform’s content. Second, more significant improvements in the teacher learning experience can be generated when the design style, tool functions, operation efficiency, and teaching methods of the platforms are optimized. These findings corroborate the previous studies that examined key factors on continuance intention in technology-assisted learning (Yang et al., 2022) and technological barriers to learning outcomes (He and Yang, 2021).

The findings of this study have crucial practical implications for developing online learning platforms for teacher professional development; however, these have some limitations in terms of generalizability. Notably, TPS-Online TPD is designed and developed primarily based on and focused on the circumstance of primary and secondary school teachers in China, which does not include other cultural backgrounds and college instructors. In addition, technology integration in teacher professional development is a long and dynamic process (Cai et al., 2019; Zhou et al., 2022). This study only measured the current teacher perceptions of the quality of online learning platforms. Thus, we suggest that future research should consider other cultures and college instructors, and periodically assess the quality of online learning platforms for teacher professional development to obtain additional accurate information and improve the use of the platform.

## Data availability statement

The original contributions presented in this study are included in the article/Supplementary material, further inquiries can be directed to the corresponding authors.

## Ethics statement

Written informed consent was obtained from the individual(s) for the publication of any potentially identifiable images or data included in this article.

## Author contributions

JZ and HY: conceptualization and writing–review and editing. JZ and ZC: methodology. JZ and WG: formal analysis. WG and ZL: investigation. BW, ZC, and HY: resources. JZ: writing–original draft preparation. BW: project administration. BW, ZC, and ZL: funding acquisition. All authors have read and agreed to the published version of the manuscript.
