# Supplementary material for: Assessing quality of online learning platforms for in-service teachers’ professional development: The development and application of an instrument
Source: Front Psychol. 2022 Oct 7;13:998196. doi: 10.3389/fpsyg.2022.998196 (PMC9585931; doi:10.3389/fpsyg.2022.998196)
Supplement: Supplementary file 1 [file Data_Sheet_1.docx]

**Appendix 1:**

Teacher perception scale of quality of online learning platforms for TPD (TPS-Online-TPD)

Platform evaluation (single choice)

1. I think the resources on the platform are updated rapidly.

○ A. Strongly agree ○ B. Agree ○ C. Undecided ○ D. Disagree ○ E. Strongly disagree

2. I would like to recommend this platform to friends and colleagues.

○ A. Strongly agree ○ B. Agree ○ C. Undecided ○ D. Disagree ○ E. Strongly disagree

3. I am very satisfied with the way the tools/sections on the webpage open, run, and jump.

○ A. Strongly agree ○ B. Agree ○ C. Undecided ○ D. Disagree ○ E. Strongly disagree

4. I think the webpage on the platform runs smoothly.

○ A. Strongly agree ○ B. Agree ○ C. Undecided ○ D. Disagree ○ E. Strongly disagree

5. The running speed of webpages on the platform and the uploading and downloading of resources are fast.

○ A. Strongly agree ○ B. Agree ○ C. Undecided ○ D. Disagree ○ E. Strongly disagree

6. It is very efficient to communicate and share resources through the platform.

○ A. Strongly agree ○ B. Agree ○ C. Undecided ○ D. Disagree ○ E. Strongly disagree

7. I think the overall page layout of the platform is reasonable.

○ A. Strongly agree ○ B. Agree ○ C. Undecided ○ D. Disagree ○ E. Strongly disagree

8. I really like the design style of the platform.

○ A. Strongly agree ○ B. Agree ○ C. Undecided ○ D. Disagree ○ E. Strongly disagree

9. The functional navigation of the web pages on the platform is clear.

○ A. Strongly agree ○ B. Agree ○ C. Undecided ○ D. Disagree ○ E. Strongly disagree

10. The colors and fonts of the platform pages are well designed.

○ A. Strongly agree ○ B. Agree ○ C. Undecided ○ D. Disagree ○ E. Strongly disagree

11. The functions and resources of the platform can support my long-term use of the platform.

○ A. Strongly agree ○ B. Agree ○ C. Undecided ○ D. Disagree ○ E. Strongly disagree

12. On the platform, I can retrieve many resources that I need for teaching.

○ A. Strongly agree ○ B. Agree ○ C. Undecided ○ D. Disagree ○ E. Strongly disagree

13. I trust the learning content and resources provided by the platform.

○ A. Strongly agree ○ B. Agree ○ C. Undecided ○ D. Disagree ○ E. Strongly disagree

14. The teaching content and Q & A provided on the platform are authoritative.

○ A. Strongly agree ○ B. Agree ○ C. Undecided ○ D. Disagree ○ E. Strongly disagree

15. The content on the platform is in line with the needs of our teachers' learning and development.

○ A. Strongly agree ○ B. Agree ○ C. Undecided ○ D. Disagree ○ E. Strongly disagree

16. The content of this learning platform is closely related to my teaching practice.

○ A. Strongly agree ○ B. Agree ○ C. Undecided ○ D. Disagree ○ E. Strongly disagree

17. After stages of learning, my teaching concepts and ideas have changed.

○ A. Strongly agree ○ B. Agree ○ C. Undecided ○ D. Disagree ○ E. Strongly disagree

18. The teaching content in the platform and the teacher's teaching are interesting enough to arouse my continuous enthusiasm for learning.

○ A. Strongly agree ○ B. Agree ○ C. Undecided ○ D. Disagree ○ E. Strongly disagree

19. The course’s learning method is suitable for my professional development needs.

○ A. Strongly agree ○ B. Agree ○ C. Undecided ○ D. Disagree ○ E. Strongly disagree

20. I am satisfied with the variety of learning methods available on the platform.

○ A. Strongly agree ○ B. Agree ○ C. Undecided ○ D. Disagree ○ E. Strongly disagree

21. Clear channels are available on the platform to help solve problems.

○ A. Strongly agree ○ B. Agree ○ C. Undecided ○ D. Disagree ○ E. Strongly disagree

22. Fast support services are available on the platform.

○ A. Strongly agree ○ B. Agree ○ C. Undecided ○ D. Disagree ○ E. Strongly disagree

23. The functional tools provided on the platform are very useful.

○ A. Strongly agree ○ B. Agree ○ C. Undecided ○ D. Disagree ○ E. Strongly disagree

24. The functional tools available on the platform are plentiful.

○ A. Strongly agree ○ B. Agree ○ C. Undecided ○ D. Disagree ○ E. Strongly disagree

25. The analysis of learning data provided by the platform is very beneficial to my learning.

○ A. Strongly agree ○ B. Agree ○ C. Undecided ○ D. Disagree ○ E. Strongly disagree

26. The platform provides personalized services, such as learning strategies and learning guidance.

○ A. Strongly agree ○ B. Agree ○ C. Undecided ○ D. Disagree ○ E. Strongly disagree

27. The quality of support services provided by the platform, such as answering questions and guidance, is high.

○ A. Strongly agree ○ B. Agree ○ C. Undecided ○ D. Disagree ○ E. Strongly disagree

**Instructions for the use of the scale**

Distribute the above scale to users of the platform under test conditions. The participant needs to be a user who has gone through the complete platform course. Within the questions, quality issues are addressed in Questions 1-11, while Questions 1-4 measure efficiency, 5-7 measure development, and 8-11 measure style. Content quality is addressed in Questions 12-20, with 12-15 measuring resources, 16 and 17 measuring effectiveness, and 18-20 measuring methods. Quality of service is evaluated in Questions 21-27, where 21 and 22 measure help, 23-25 measure functional tools, and 26 and 27measure guidance. The value of each dimension is the product of the average score of the dimension and the weight value. The weight values are shown in Table 5.
