# Supplementary material for: Assessing quality of online learning platforms for in-service teachers’ professional development: The development and application of an instrument
Source: Front Psychol. 2022 Oct 7;13:998196. doi: 10.3389/fpsyg.2022.998196 (PMC9585931; doi:10.3389/fpsyg.2022.998196)
Supplement: Supplementary file 2 [file Data_Sheet_2.docx]

**Appendix 2:**

Expert background information form

| **Number** | **Category** | **Gender** | **Professional background** | **Working experience** |
| --- | --- | --- | --- | --- |
| 1 | College instructor | Male | Teacher professional development in the information environment | 21 |
| 2 | College instructor | Male | Teacher preparation in information technology environment | 32 |
| 3 | College instructor | Male | Technology-enabled classroom and online learning platform development | 27 |
| 4 | College instructor | Female | Smart learning environments and online learning platform development | 22 |
| 5 | College program advisor | Male | Online learning | 15 |
| 6 | College program advisor | Male | Teacher preparation and professional development | 25 |
| 7 | School teacher | Female | Teacher training project planning | 11 |
| 8 | School teacher | Female | Information technology integration | 9 |
| 9 | Subject supervisor | Female | Geography teaching supervision and information teaching guidance | 30 |
| 10 | Subject supervisor | Female | Mathematics teaching supervision and informationized teaching guide | 21 |
| 11 | Subject supervisor | Female | English teaching supervision and information teaching guidance | 18 |
| 12 | Subject supervisor | Male | History teaching supervision and information teaching guidance | 19 |
| 13 | School teacher | Male | Mentorship for new Chinese teachers | 31 |
| 14 | School teacher | Male | Mentorship for new biology teachers | 37 |
